# Radiotherapy for Keloids: A Comprehensive Narrative Review

**DOI:** 10.7759/cureus.93869

**Published:** 2025-10-05

**Authors:** Hsin-Hua Nien, Pei-Chieh Yu, Yu-Hsiu Yen, Yu-Lun Tsai, Li-Ying Wang, Chen-Hsi Hsieh

**Affiliations:** 1 Department of Radiation Oncology, Cathay General Hospital, Taipei, TWN; 2 School of Medicine, Fu Jen Catholic University, Taipei, TWN; 3 School and Graduate Institute of Physical Therapy, National Taiwan University College of Medicine, Taipei, TWN; 4 Division of Radiation Oncology, Department of Radiology, Far Eastern Memorial Hospital, Taipei, TWN

**Keywords:** brachytherapy, electron, external beam radiotherapy, keloid, photon, radiotherapy

## Abstract

Keloids cause uncomfortable symptoms that disrupt daily life. The outcomes of monotherapy are often unsatisfactory. However, surgical excision followed by adjuvant radiotherapy has been shown to reduce recurrence rates. Radiotherapy for keloid treatment can be administered using various radiation modalities and sources. Each radiation modality and source has unique treatment properties and requires careful setup considerations. Treatment parameters and variability in clinical outcomes and adverse effects have been reported across studies.

This literature review aimed to provide a narrative overview of the differences and defining characteristics of each radiation modality used in keloid management and to summarize the clinical evidence supporting their treatment efficacy. Radiation treatment regimens, recurrence rates, and adverse effects were summarized according to modality. The factors affecting treatment efficacy were analyzed. A comprehensive understanding of each radiation modality’s properties enables the development of institution-specific treatment strategies, tailored to available resources and supported by multidisciplinary collaboration. Cautious planning and delivery of radiotherapy, combined with surgery, can result in favorable clinical outcomes for patients with keloids.

## Introduction and background

Keloids develop at sites of previous dermal injury and are often associated with pain, pruritus, and complications such as ulceration and bleeding [1,2]. They may arise following trauma, piercings, surgical wounds, acne, and other skin insults [1,3,4]. Wound healing is a complex process. Disruptions in regulatory signaling have been implicated in the formation of hypertrophic scars and keloids. In keloid tissue, excessive fibroblast proliferation and an increased Type I to Type III collagen ratio have been observed [5].

Keloids commonly develop in the chest, abdomen, shoulders, upper back, neck, and earlobes [6]. Their incidence has been reported to vary by race [4,7-10]. Regardless of racial background, a higher incidence has been observed among younger individuals, women, and those with a positive family history [6-8,11]. Predisposing factors are broadly categorized into genetic, systemic, and local factors. Specific gene sequences have been identified in association with keloid formation [12-15], and certain single-nucleotide polymorphisms have been correlated with keloid severity [16-18]. Systemic contributors, such as hypertension [19], inflammatory cytokines [20], sex hormones, and pregnancy [21-24], have been found to exacerbate keloid development. In addition, local mechanical tension has been implicated as a contributing factor [25].

The presentation and severity of keloids must be evaluated. Several scar assessment scales are commonly employed for this purpose [26-28], each offering distinct definitions of scar characteristics. Hsueh et al. evaluated preoperative keloid conditions using the Vancouver Scar Scale (VSS) and the Japan Scar Workshop Scar Scale (JSS). They reported a strong correlation between both scales and keloid recurrence. Notably, the JSS was found to independently predict the risk of keloid recurrence [29]. The components of each scar evaluation scale are summarized in Table 1.

**Table 1:** Comparison of evaluation components across scar evaluation scales. *Patient assessment included eight detailed questions, each scored on a scale from 1 to 10. SBSES, Stony Book Scar Evaluation Scale; MSS, Manchester Scar Scale; JSS, Japan Scar Workshop Scar Scale; POSAS, Patient and Observer Scar Assessment Scale

	Vancouver	SBSES	MSS	JSS	POSAS
Scar presentation					
Vascularity	+				+
Pigmentation/color	+	+	+		+
Pliability	+				+
Height	+	+		+	+
Width		+		+	
Hatch marks/suture marks		+			
Overall appearance		+			
Matte vs. shiny			+		
Contour			+		
Distortion			+		
Texture			+		
Size (cm^2^)				+	
Characteristic shape				+	
Erythema around scars				+	
Other than scar presentation					
Scar number				+	
Onset region				+	
Age at onset				+	
Scar causes				+	
Frequency of subjective symptoms				+	
Human race				+	
Familial tendency				+	
Relief					+
Patient assessment*					+
Score	0-13	0-5	5-18	0-25 (Classification); 0-18 (Induration)	6-60 (Patient); 6-60 (Observer)

Several methods have been used in the management of keloids, including surgical incision, corticosteroid injections, carbon dioxide laser therapy, Nd:YAG laser treatment, silicone gel application, retinoic acid, silicone sheet coverage, immunomodulators, cryotherapy, and radiotherapy. Among these, surgical resection directly removes keloid tissue and plays a major role in treatment. However, the control rates achieved by monotherapies have been unsatisfactory, ranging from 45% to 100% [6,30-32]. Surgery followed by radiotherapy is an effective treatment for keloids, offering favorable local control and symptom relief. The mechanism of radiotherapy in keloid control involves multiple biological effects. Radiation-induced DNA damage inhibits fibroblast proliferation, while angiogenesis is attenuated because the developing endothelial vascular bed is radiosensitive. In addition, radiotherapy modulates gene expression and signaling pathways related to the microtubule-associated complex, extracellular matrix organization, and oxidative phosphorylation. These biological responses collectively reduce collagen production, induce cell apoptosis, and suppress cell viability [33-35]. Nevertheless, radiation delivery modalities vary across institutions, each with unique characteristics. The reported rates of keloid control and adverse effects are dependent on the specific modality and treatment parameters. This study aimed to provide a comprehensive review of recent advancements in radiotherapy for keloids and summarize the clinical evidence supporting the treatment efficacy of each modality, according to the current literature.

## Review

Materials and methods

Data Source and Search Strategy

Literature published between January 1, 2004, and March 1, 2024, was retrieved from PubMed and Web of Science. The Patient (keloids), Intervention (radiotherapy), Comparison (different radiation modalities), and Outcomes (recurrence rate and adverse effects of radiation therapy) framework was used for formulating the clinical questions and search strategy. The search query required the keywords “keloid” in combination with at least one of the following terms: “radiotherapy,” “electron,” “photon,” or “brachytherapy.” The exact search string syntax applied was: ("keloid"[Title] OR "keloids"[MeSH Terms]) AND ("radiotherapy"[MeSH Terms] OR "radiotherapy"[Title] OR "electron"[Title] OR "photon"[Title] OR "brachytherapy"[Title]) AND ("2004/01/01"[Date - Publication] : "2024/03/31"[Date - Publication]). All records were imported into Zotero for reference management, including removing duplicates.

Eligible studies included original articles and meta-analyses focusing on radiotherapy or adjuvant radiotherapy for keloids. Exclusion criteria were review articles, case reports, studies unrelated to radiotherapy or adjuvant radiotherapy, non-human research, and articles not published in English. Considering the heterogeneity in trial nature, follow-up duration, and treatment parameters, we systematically extracted data on study design, radiation modality, total dose and fractionation, recurrence rate, and reported adverse effects from each study to ensure the quality and comprehensiveness of this narrative review. These variables were then descriptively summarized to highlight key patterns and findings. As this study involved only a literature review of previously published human research, ethical committee approval was not required.

Results

**Figure 1:**
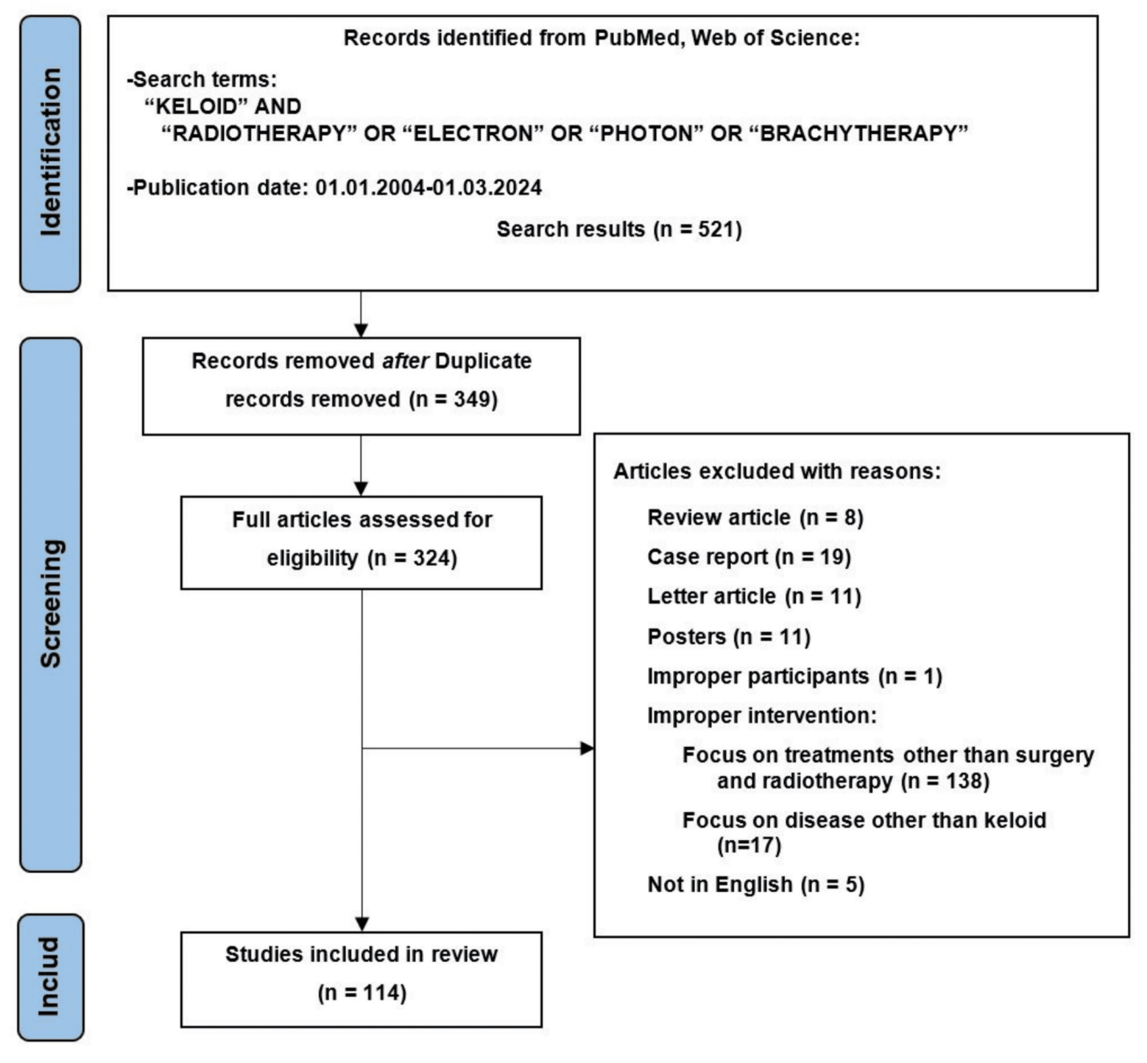
Preferred Reporting Items for Systematic Reviews and Meta-Analyses diagram of the literature review and article selection process.

A total of 521 articles were retrieved from the two electronic databases. After removing duplicates, 349 studies were included. Following a detailed review, additional exclusions were made: 8 review articles, 19 case reports, 11 letters, 11 posters, 5 non-English articles, and 179 studies with irrelevant topics, including those involving treatment modalities beyond surgery and radiotherapy. Ultimately, 114 eligible studies were included in this review, as shown in Figure 1.

Electronic databases, including PubMed and Web of Science, were screened. The Population, Intervention, Comparison, and Outcome structure was applied for formulating the clinical question and guiding the search strategy.

Factors influencing radiotherapy outcomes

Several factors may influence the effectiveness of radiotherapy. Radiotherapy dosage plays a critical role. The total delivered dose and daily fraction size both influence the treatment outcomes and adverse effects. Radiation dose distribution varies according to the type of radiation source and radiation energy. Each source and energy level requires different treatment parameters for optimal dose delivery. By contrast, the delivery applicators and settings determine the preparation time and interval between surgery and the initiation of radiotherapy.

Dosage evaluation of radiotherapy

Most radiotherapy regimens employ a fractionated schedule to balance normal tissue tolerance with effective tumor or target cell damage. The biological effectiveness of a fractionated radiotherapy schedule is commonly assessed using a biologically effective dose (BED). The BED is determined by three main factors: the number of fractions (n), the dose per fraction (d), and the α/β ratio, which reflects the tissue's sensitivity to fractionation. Based on clinical observations, tissues composed of cells with high metabolic activity and rapid turnover - such as cancer and skin cells - are classified as early-responding tissues, typically characterized by an α/β ratio of approximately 10 Gy. Conversely, late-responding tissues, such as muscle, tend to exhibit lower α/β ratios, ranging from 1 to 4 Gy depending on the specific cell type. Generally, late-responding tissues are more sensitive to larger doses per fraction compared with early-responding tissues.

As BED values vary depending on the α/β ratio used in the calculation, the results are expressed in the format BED(α/β) to ensure clarity. For example, BED10 indicates that the BED was calculated using an α/β ratio of 10 Gy. The formula for calculating BED is as follows: $\text{BED} = nd \left(1 + \frac{d}{\alpha/\beta} \right)$.

Kal et al. suggested an α/β ratio of 10 Gy, classifying keloids as an early-responding tissue. In their study, various postoperative radiotherapy regimens were converted into BED. A BED of ≥30 Gy (using α/β = 10) was found to be associated with a recurrence rate of less than 10% [36,37]. However, Flickinger suggested an α/β ratio of approximately 2.08 Gy, indicating that keloids may behave more like late-responding tissue. Under this assumption, a higher dose per fraction may improve local control, and extending the overall treatment time may offer no additional benefit [38]. Comparable control rates were observed with BED2 values ranging from 60 to 70 Gy [39,40]. Commonly used protocols for adjuvant radiotherapy are listed in Table 2. BED serves as a valuable tool for evaluating the effects of changes in fraction size and total dose. However, the choice of α/β ratio significantly affects BED conversion, as shown in Table 3. As the current literature has not reached a consensus on the appropriate α/β ratio for keloids, with studies using both 2 and 10 Gy, this review includes BED calculations using both values, reported as BED2 and BED10.

**Table 2:** Keloid radiotherapy regimens reported in the literature over the past 20 years, including daily dose per fraction and the total number of fractions used. Annotation markers: ^a^ Both BED10 and BED2 estimation reach effective treatment efficacy (BED10 ≧ 30 and BED2 ≧ 60); ^b^ Inconsistency of estimated effective treatment efficacy between BED10 and BED2; V represents the presence of the corresponding dose-fractionation schedule reported in the literature. BED, biologically effective dose; BED2, biologically effective dose calculated with α/β ratio 2 Gy; BED10, biologically effective dose calculated with α/β ratio 10 Gy

Fraction size	1 fraction	2 fractions	3 fractions	4 fractions	5 fractions	Composite dose schedule
3 Gy	-	-	-	V	V	-
4 Gy × 1 fraction + 3 Gy × 2 fractions	-	-	-	-	-	V
4 Gy	-	-	V	V	^b^ V	-
6 Gy × 1 fraction + 5 Gy × 2 fractions	-	-	-	-	-	V
5 Gy	-	-	V	^a^ V	-	-
6 Gy	-	V	^b^ V	-	-	-
7.5 Gy	-	V	-	-	-	-
8 Gy	V	-	-	-	-	-
9 Gy	-	^a^ V	-	-	-	-
10 Gy	^b^ V	-	-	-	-	-
12 Gy	^b ^V	-	-	-	-	-
13 Gy	^a^ V	-	-	-	-	-

**Table 3:** Correlated BED to according to the radiotherapy protocols. Annotation markers: ^a^ Both BED10 and BED2 estimation reach effective treatment efficacy (BED10 ≧ 30 and BED2 ≧ 60); ^b^ Inconsistency of estimated effective treatment efficacy between BED10 and BED2. BED2, biologically effective dose calculated with α/β ratio 2 Gy; BED10, biologically effective dose calculated with α/β ratio 10 Gy

Fraction size	1 fraction	2 fractions	3 fractions	4 fractions	5 fractions	6 fractions
3 Gy	BED10 = 3.9; BED2= 7.5	BED10 = 7.8; BED2= 15.0	BED10 = 11.7; BED2= 22.5	BED10 = 15.6; BED2= 30.0	BED10 = 19.5; BED2 = 37.5	BED10 = 23.4; BED2 = 45.0
4 Gy	BED10 = 5.6; BED2= 12.0	BED10 = 11.2; BED2= 24.0	BED10 = 16.8; BED2= 36.0	BED10 = 22.4; BED2= 48.0	^b^ BED10 = 28.0; BED2= 60.0	^a^ BED10 = 33.6; BED2= 72
5 Gy	BED10​​​​​​​ = 7.5; BED2= 17.5	BED10​​​​​​​ = 15.0; BED2= 35.0	BED10​​​​​​​ = 22.5; BED2= 52.5	^a^ BED10​​​​​​​ = 30.0; BED2= 70.0	^a^ BED10​​​​​​​ = 37.5; BED2= 87.5	^a^ BED10​​​​​​​ = 45.0; BED2= 105.0
6 Gy	BED10​​​​​​​ = 9.6; BED2= 24.0	BED10​​​​​​​ = 19.2; BED2= 48.0	^b^ BED10​​​​​​​ = 28.8; BED2= 72.0	^a^ BED10​​​​​​​ = 38.4; BED2= 96	^a^ ​​​​​​​BED10​​​​​​​ = 48.0; BED2= 120.0	^a^ ​​​​​​​BED10​​​​​​​ = 57.6; BED2= 144.0
8 Gy	BED10 = 14.4; BED2= 40.0	^b^ ​​​​​​​BED10 = 28.8; BED2= 80.0	^a^ BED10 = 43.2; BED2= 120.0	^a^ ​​​​​​​BED10 = 57.6; BED2= 160.0	-	-
9 Gy	BED10 = 17.1; BED2= 49.5	^a^ ​​​​​​​BED10 = 34.2; BED2= 99.0	^a^ ​​​​​​​BED10 = 51.3; BED2= 148.5	-	-	-
10 Gy	^b^ ​​​​​​​BED10 = 20.0; BED2= 60.0	^a^ ​​​​​​​BED10 = 40.0; BED2= 120.0	-	-	-	-
11 Gy	^b^ ​​​​​​​BED10 = 23.1; BED2= 71.5	^a^ ​​​​​​​BED10 = 46.2; BED2= 143.0	-	-	-	-
12 Gy	^b^ ​​​​​​​BED10 = 26.4; BED2= 84.0	^a^ ​​​​​​​BED10 = 52.8; BED2= 168.0	-	-	-	-
13 Gy	^b^ ​​​​​​​BED10 = 29.9; BED2= 97.5	^a^ BED10 = 59.8; BED2= 195.0	-	-	-	-
16 Gy	^a^ BED10 = 41.6; BED2= 144.0	^a^ ​​​​​​​BED10 = 83.2; BED2= 288.0	-	-	-	-

Radiotherapy modalities applied to keloid treatment

**Figure 2:**
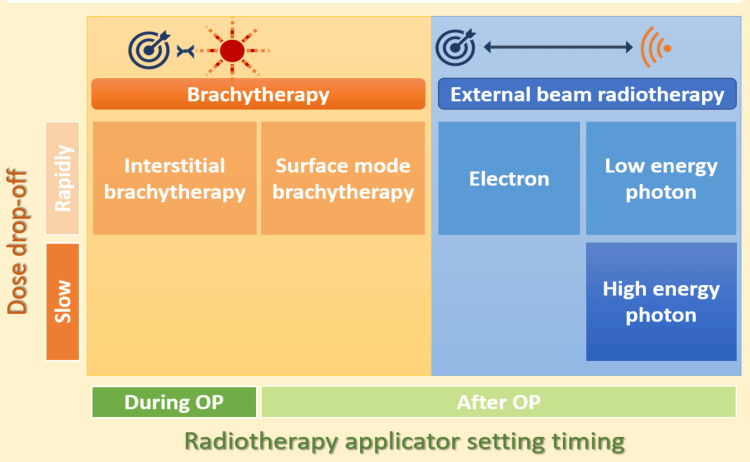
Categories of radiotherapy applied in keloid treatment. OP, operation

Radiotherapy for keloids can be broadly classified into external beam radiotherapy (EBRT) and brachytherapy. The fundamental principle of brachytherapy involves placing radioactive sources near the target tissue, thereby delivering a high radiation dose directly to the treatment site. Unlike brachytherapy, EBRT delivers radiation from a distance, with the beam penetrating surrounding tissues to reach the target. Radiation energy influences both the depth of tissue penetration and the steepness of the dose gradient, necessitating tailored treatment parameters for each type of radiotherapy. Keloid radiotherapy can be broadly categorized, as illustrated in Figure 2.

Radiotherapy can be classified as brachytherapy and EBRT, based on the distance between the target keloid excision wound and the radiation source. Radiation energy and particle type greatly influence dose distribution. High-energy photons penetrate deeply, with a slow dose fall-off. Conversely, brachytherapy, low-energy photons, and electrons exhibit rapid dose fall-off. Interstitial brachytherapy applicators are placed directly in the excision wound and irradiate during or immediately after surgery, starting intraoperatively. Other radiotherapy modalities require postoperative planning based on wound information.

Brachytherapy

Brachytherapy can be administered in two modes: interstitial brachytherapy and surface-mode brachytherapy. Brachytherapy is further categorized into high-dose-rate (HDR) and low-dose-rate (LDR) modalities. HDR brachytherapy typically involves fractionated treatment, with a minimum interval of six hours between sessions. The activity of LDR iridium 192 (192Ir) sources ranges from 0.2 mCi to 15 mCi per seed, approximately 1/10,000 of the HDR seed activity. Modern HDR brachytherapy is typically performed using remote afterloading systems. Radioactive sources are delivered into the catheters only during treatment sessions; no radiation exposure occurs between treatments. However, LDR brachytherapy involves continuous irradiation, often necessitating patient admission to a shielded room for 36-72 hours.

HDR Brachytherapy: Interstitial Brachytherapy Setting and Target

Interstitial brachytherapy delivers radiation through a source inside the catheter. Patients typically undergo surgical excision of keloid tissue. Following resection, brachytherapy catheters are usually placed 0.5 cm below the skin, with the catheter ends exiting through the sutured wound. The prescribed dose depth generally ranges from 4 to 7 mm, depending on the catheter placement depth [41]. Although a 0.5-cm margin around the catheter is commonly defined as the treatment target, Barragán et al. and Anderson et al. delineated high-risk clinical targets on computed tomography simulation images, contouring 0.3 to 0.5 cm of skin thickness below the scar for dose assessment [42,43]. For catheters placed deeper than 0.5 cm, Bijlard et al. extended the target margin to a maximum of 0.7 cm to ensure adequate dose coverage [44]. 192Ir is the most commonly used source in brachytherapy. However, cobalt-60 (60Co) may also be used for interstitial applications [42,45].

HDR Brachytherapy: Interstitial Brachytherapy Dose and Recurrence Rate

The treatment schedules for HDR brachytherapy vary, with reported recurrence rates ranging from 0% to 44%, depending on the specific radiation regimen. Improved local control has been associated with higher total doses and larger doses per fraction [41-57]. The reported HDR brachytherapy protocols and their corresponding associated recurrence rates are listed in Table 4.

**Table 4:** Recurrence rate reported according to radiotherapy regimens of high-dose rate brachytherapy. Annotation markers: ^a^ Both BED10 and BED2 estimation reach effective treatment efficacy (BED10 ≧ 30 and BED2 ≧ 60); ^b^ Inconsistency of estimated effective treatment efficacy between BED10 and BED2; * Denotes earlobe. BED, biologically effective dose; BED2, biologically effective dose calculated with α/β ratio 2 Gy; BED10, biologically effective dose calculated with α/β ratio 10 Gy

Fraction size	1 fraction	2 fractions	3 fractions	4 fractions	5 fractions
3 Gy	-	-	-	38% [49], 26.7% [56]	-
4 Gy × 1 + 3 Gy × 2	-	-	44% [48]	-	-
4 Gy	-	-	4.9% [42], 20% [54], 21.43% [46]	-	-
6 Gy × 1 + 4 Gy × 2	-	-	3% [48]	-	-
5 Gy	-	-	2% [45], 12% [50], *8% [51]	-	-
6 Gy	-	3.1% [52], 25% [44]	^b^ 0% [48], 6% [47], 6.3% [57], 8% [53], 8% of head [47], 3% of trunk [47], 25% of limb injuries [47], 23.4% [44]	-	-
8 Gy	0% [43]	-	-	-	-
9 Gy	-	^a^ 22.1% [44]	-	-	-
13 Gy	^b^ 29.2% [41]	-	-	-	-

HDR Brachytherapy: Surface Mode Brachytherapy Setting and Target

Unlike interstitial brachytherapy, in which radioactive sources are inserted into tissue, surface-mode brachytherapy involves the placement of radioactive sources directly over a closed wound. Radiation is delivered postoperatively through molds placed on the skin. Delivery applicators include acrylic molds [58], Leipzig applicators [58], integrated strontium-90 (90Sr) surface applicators [59], and three-dimensional-printed conformal devices [60,61]. The radiation target in surface-mode brachytherapy typically extends to a depth of 0.5 cm beneath the skin for general anatomical sites. For lesions located on the earlobe with a thickness of <5 mm, or for surgical wounds on the ear helix, surface-level dose prescriptions are commonly used [58]. The radioactive sources used include 192Ir and 90Sr. The radiation dose from 90Sr is highly superficial, with its dose falling to nearly zero at 0.5 cm below the skin surface - substantially shorter than the dose penetration range of 192Ir.

HDR Brachytherapy: Surface Mode Brachytherapy Dose and Recurrence Rate

Iridium 192 (192Ir): The recurrence rate of surface-mode brachytherapy using 192Ir ranges from 0% to 42.8% [58,62,63]. Ahmad et al. compared the response rates of different surface-mode 192Ir brachytherapy regimens. Their study demonstrated a high recurrence rate of 42.8% following a single fraction of 8 Gy, whereas more favorable outcomes were observed with other regimens, including 10 Gy × 1 fraction, 5 Gy × 3 fractions, and 6 Gy × 3 fractions [63]. The reported treatment protocols for surface-mode 192Ir brachytherapy and the associated recurrence rates are listed in Table 5.

**Table 5:** Recurrence rate reported according to radiotherapy regimens of surface mode 192Ir brachytherapy. Annotation markers: ^a^ Both BED10 and BED2 estimation reach effective treatment efficacy (BED10 ≧ 30 and BED2 ≧ 60); ^b^ Inconsistency of estimated effective treatment efficacy between BED10 and BED2. BED, biologically effective dose; BED2, biologically effective dose calculated with α/β ratio 2 Gy; BED10, biologically effective dose calculated with α/β ratio 10 Gy; 192Ir, Iridium 192

Fraction size	1 fraction	2 fractions	3 fractions	4 fractions	5 fractions	6 fractions
5 Gy	-	-	5% [58], 0% [62], 15% [63]	^a^ 9.7% [62]	-	-
6 Gy	-	-	^b^ 10.5% [63]	-	-	-
8 Gy	42.8% [63]	-	-	-	-	-
10 Gy	^b^ 10.5% [63]	-	-	-	-	-

Stronitum-90 (90Sr): 90Sr/yttrium-90 (90Y) has also been used in surface-mode brachytherapy. Radiation is delivered via β-particle emission, as 90Sr decays into 90Y, which exhibits significantly lower tissue penetration compared with 192Ir, photon, and electron radiotherapy. The radiation dose rapidly decreases to approximately 1%-3% at a tissue depth of 5 mm [64]. Approximately 75% of the β-radiation is absorbed within the first 2 mm below the skin surface, with the remaining dose absorbed within the subsequent 1 mm of tissue [65]. 

Fraunholz et al. reported outcomes from postoperative radiotherapy using 90Sr in 83 keloids from 66 patients. A total dose of 20 Gy was delivered in four daily fractions, with 75% of patients receiving their first irradiation on the day of surgery. The recurrence rate was reported as 36% [64]. Viani et al. analyzed data from 612 patients with keloids treated with postoperative 90Sr at a dose of 20 Gy in 10 daily fractions. The interval between surgery and radiotherapy varied from 2 to 168 hours, with a median of 44 hours. The recurrence rates were 11.9% for patients treated within 24 hours post-excision and 13.1% for those treated after 24 hours [65]. Wagner et al. reported recurrence rates ranging from 8% to 33%, depending on the total radiation dose, which varied between 7 and 28.5 Gy [59].

Adverse Effects of HDR Interstitial Brachytherapy

**Figure 3:**
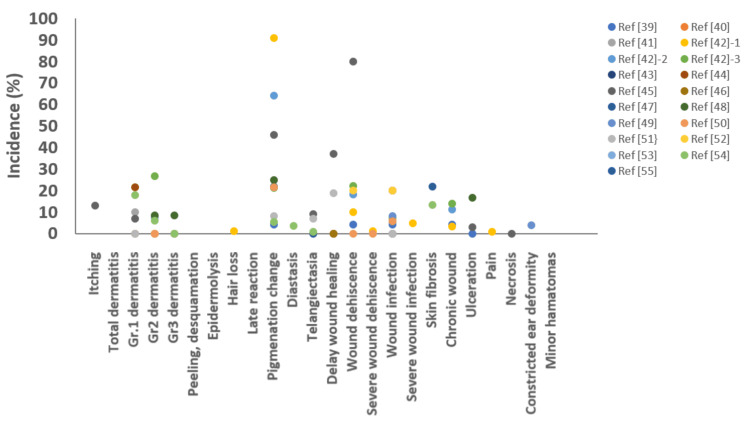
Incidence rates of adverse effects following high-dose-rate (HDR) interstitial brachytherapy reported in literature over the past 20 years. Each data point represents the incidence rate of adverse effects reported in an individual study.

Brachytherapy is characterized by the delivery of an extremely high radiation dose near the radioactive source, with a rapid dose fall-off. The dose administered near the source can be two to four times greater than that administered at a distance of 0.5 cm from the source [42,51]. The acute adverse effects associated with interstitial brachytherapy include erythema and desquamation (7%-21.4%) [43,46,47,56], itching (5.9%-13.0%) [47,56], and Grade 2 dermatitis (0%-26.7%) [44,50]. Grade 3 dermatitis has been observed in 8.3% of patients [50]. The reported chronic complications include increasing post-treatment pain [44]; alopecia (1.2%) [44]; pigmentation change (1.2%-61.7%) [41,44,47,49,50,52,53,56,57]; diastasis (3.5%) [56]; telangiectasia (0.9%-9%) [47,53,56]; delayed wound healing (0%-37%) [41,42,44,47,53]; wound dehiscence (4.2%-22.1%) [41,44,54]; minimal suture release (80.0%) [47]; severe wound dehiscence (1.2%) [44]; wound infection (0%-20.0%) [41,42,44,47,51,52,54]; severe wound infection (4.7%) [44]; ulceration (3%-16.7%) [47,50]; and skin fibrosis (13.2%-22.0%) [49,56]. The adverse effects associated with HDR interstitial brachytherapy reported across the study are shown in Figure 3.

Adverse Effects of HDR Brachytherapy: Surface Mode Brachytherapy - 192Ir

The common adverse effects of surface mold brachytherapy include pruritus and skin erythema. Fraunholz et al. reported that 87% of the patients experienced skin alterations, including mild (17.0%), moderate (33%), and severe (37%) changes. A higher incidence of skin alterations was observed in patients without recurrence, including acute erythema (21%-24%), telangiectasia (10.4%-48%), hypopigmentation (11%-63%), and hyperpigmentation (42%) [59,64,65]. Keloids resulting from burns were associated with a lower rate of favorable cosmetic outcomes compared with other etiologies (46.2% vs. 75.2%) [65].

Dose evaluation must also account for the presence of a spacer between the skin and the applicator [62]. Reported acute reactions included erythema in nearly all patients [62], with Grade 1 and 2 radioepithelitis observed in 15.0% and 1.0% of patients, respectively [58]. Chronic reactions included Grade 1 hypopigmentation (27.5%) [58], Grade 1 fibrosis in the center of scars (22.5%) [58], and wound complications (4.8%) [62]. The utilization of Leipzig applicators has been associated with a higher incidence of radioepithelitis, and the radiation dose at a 0.5-cm tissue depth has been significantly associated with the development of chronic toxicities [58].

LDR Brachytherapy: Interstitial Brachytherapy

The total dose of LDR brachytherapy used as adjuvant radiotherapy in patients with keloids generally ranges from 16 to 20.5 Gy [49,66,67]. Arnault et al. administered LDR brachytherapy at an average dose of 17.9 ± 2.2 Gy over an irradiation period of 44.3 ± 11.3 h, reporting a recurrence rate of 23.6% [67]. De Cicco et al. delivered a mean dose of 16 Gy, with a recurrence rate of 30.4% [49].

Adverse Effects of LDR Interstitial Brachytherapy

The use of LDR interstitial brachytherapy for keloid treatment has been reported less frequently over the past two decades compared with the use of other treatment modalities. The documented adverse effects of LDR interstitial brachytherapy include telangiectasia (15.2%-72.0%) [49,67], depigmentation (32.6%-67.0%) [49,67], and sclerosis (31.0%) [67].

External beam radiotherapy (EBRT)

*Electron Beam Radiotherapy*​​​​​​​*: Megavoltage (MV) Electron*

MV electron beam radiotherapy is suitable for superficial irradiation. A skin-sparing effect is typically observed, resulting in only 80%-90% of the prescribed dose being delivered to the skin surface. This relatively low surface dose presents a limitation when compared with that of other radiation modalities. Thus, a bolus is frequently applied to the target skin surface in clinical practice to enhance surface dosing. Similarly, wax has been utilized in various studies for the same purpose [68-70]. In addition to the use of bolus and wax, Vila Capel et al. proposed the placement of a 4-mm aluminum spoiler above the electron applicator to ensure an adequate surface dose [71]. Considering the constricted nature of electron dose distribution, the treatment field is recommended to extend 1-2 cm beyond the surgical scar to ensure complete coverage of the targeted area. Cerrobend blocks were fabricated for electron beam shielding based on the designed treatment field to protect surrounding tissue, and they typically require several hours to produce.

EBRT: MV Electron Radiotherapy, Dose, and Recurrence Rate

The reported recurrence rate following EBRT ranges from 0% to 56.5% [2,29,55,69-94]. Recurrence rates are influenced by factors such as lesion location, radiotherapy regimens, and the timing of postoperative irradiation (Table 6). Lesions located on the earlobe exhibit the lowest recurrence rates (0%-6.9%), whereas those on the chest, shoulder, and suprapubic regions demonstrate rates as high as 50.8%. Higher recurrence has also been observed in auricular lesions, excluding the earlobe [73,78].

**Table 6:** Recurrence rate reported according to radiotherapy regimens of electron beam radiotherapy. Annotation markers: ^a^ Both BED10 and BED2 estimation reach effective treatment efficacy (BED10 ≧ 30 and BED2 ≧ 60); ^b^ Inconsistency of estimated effective treatment efficacy between BED10 and BED2. * Earlobe; ** Auricle excluding earlobe; *** Umbilicus; **** High-risk recurrence region: anterior chest wall, scapular region, suprapubic region. ^T1^ Radiotherapy initiated within 24 h after surgery; ^T2^ Radiotherapy initiated within 24-48 h after surgery; ^T3^ Radiotherapy initiated within 48 h after surgery; ^T4^ Radiotherapy initiated within 72 h after surgery. RT: radiotherapy; IORT: intraoperative radiotherapy; BED, biologically effective dose; BED2, biologically effective dose calculated with α/β ratio 2 Gy; BED10, biologically effective dose calculated with α/β ratio 10 Gy

Fraction size	1 fraction	2 fractions	3 fractions	4 fractions	5 fractions
3 Gy	-	-	-	-	Refractory keloid 23.5% [71]
4 Gy	-	-	35.7% (RT at 0, 2, and 4 days after surgery) [89]	15% [75]	^b^ 10% [77]; 91.5%, *7.32% [93]
4.5 Gy	-	-	20% [2]	-	-
5 Gy	-	*0%-6.9% [73]	6% [80], 13.3% [68], 13.6% [78], 14.3% (ear), *5.7%, **9.4%-38.5%, 16.7% (neck), 26.7% (upper limb), 9.1% (lower limb) [73], ****36.4%-43.1% [73], 35% [79]	^a^ ****11.4%-23.1% [73]	-
6 Gy	-	-	^b^ ****7.1%–16.7% [73], 10.6% [81], 5.3% [94]	-	-
7.5 Gy	-	^b^ **4.5% [73], 10.4% [73], ***8.8% [91]	-	-	-
8 Gy	*6.7% [73] ^T4^, 13.8% [82] ^T1^, 56.5% [74]	-	-	-	-
9 Gy	-	^a^ ​​​​​​​With 1 week interval between the 2 fractions: ^T1 ^8.52%, ^T2 ^13.96%, ^T3 ^18.78 [69]	-	-	-
10 Gy	^b^ ​​​​​​​^T4^ 0% [83], *0% [85], ****8.75% [84]	-	-	-	-
12 Gy	^b^ ​​​​​​​9.5% IORT [86]	-	-	-	-

EBRT: MV Electron Radiotherapy and Adverse Events

**Figure 4:**
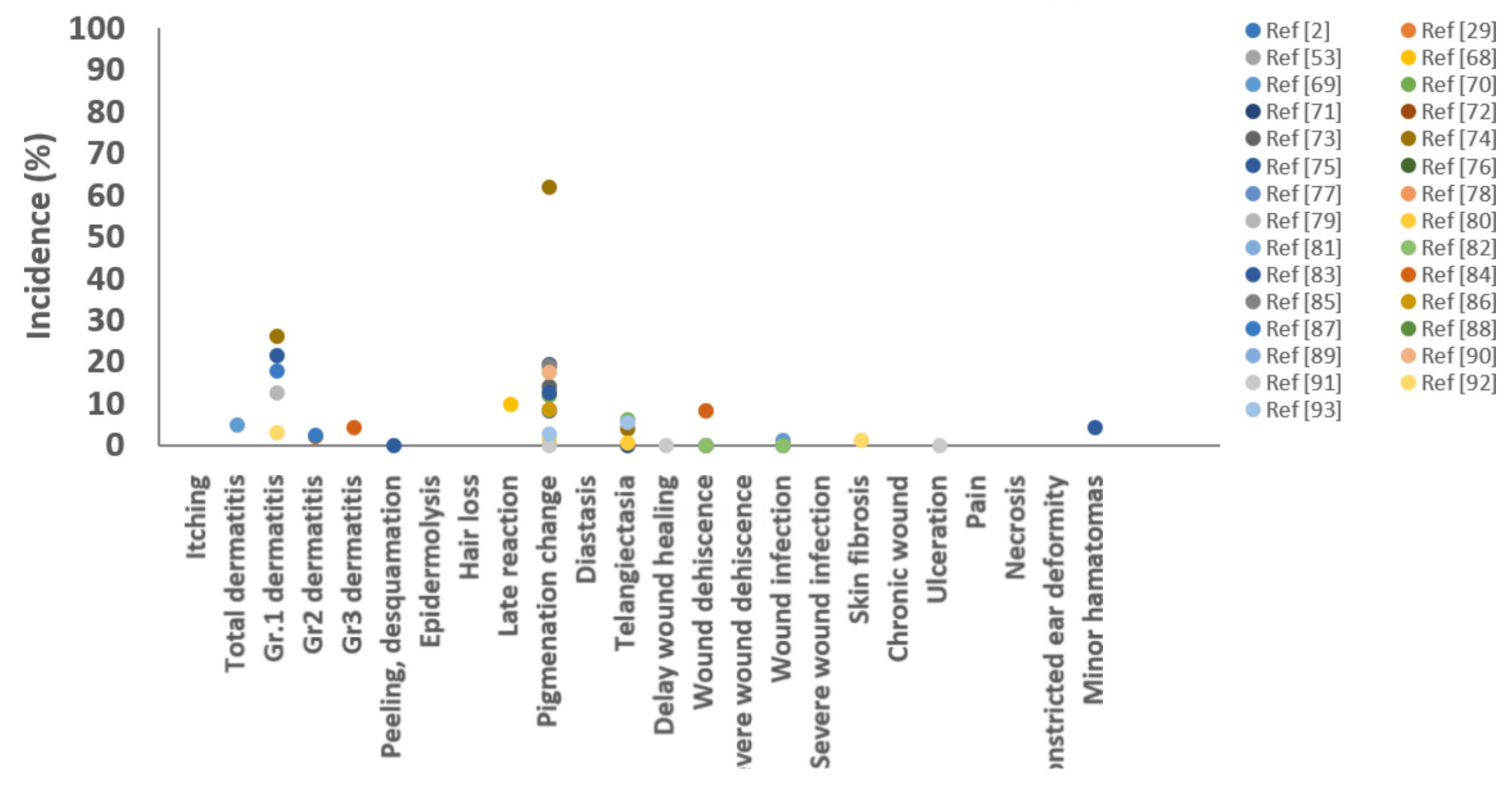
Incidence rates of adverse effects following electron beam radiotherapy reported in literature over the past 20 years. Each data point represents the incidence rate of adverse effects reported in an individual study.

The adverse effects associated with electron radiotherapy for keloid treatment are generally mild: erythema in 17% [75]; Grade 2 and Grade 3 radiation dermatitis in 2.1%-2.2% [85,88] and 4.2%, respectively [85]; minor hematoma in 4.2% [85]; skin pigmentation changes in 0%-80.9% [68,69,71,72,74,75,78,81,84,86-88,91,92,94]; telangiectasia in 0.5%-6% [71,75,81,94]; wound dehiscence in 8.1%-12.5% [82,84,85]; and wound infection in 1.2% (one patient) [70]. The adverse effect rates reported across studies are illustrated in Figure 4.

EBRT: Electron Radiotherapy - Kilovoltage (kV) Electron

Among the reviewed studies, van de Kar et al. were the only group that applied kilovoltage (kV) electrons as adjuvant radiotherapy for keloid treatment. A recurrence rate of 71.9% was reported following a prescribed dose of 12 Gy, delivered in three to four fractions [95]. As previously discussed, the dose penetration and steep dose gradient of MV electrons make them suitable for superficial lesions, with the dose rapidly decreasing to zero within a few centimeters. However, the penetration depth is significantly reduced when the electron energy is lowered to the kV range. Although the study did not provide a dose distribution profile for kV electrons, the limited penetration may have contributed to the high recurrence rate.

EBRT: High-Energy Photon Radiotherapy, Dose, and Recurrence Rate

Malaker et al. delivered 16 Gy in four daily fractions to 47 earlobe keloids, achieving a control rate of 87.2% [96]. Lin et al. delivered 13.5 Gy over three consecutive days, reporting a one-year control rate of 81.8%, compared with 63.4% in the electron beam group within the same study [2]. Abdus-Salam et al. administered adjuvant radiotherapy at a dose of 12 Gy, delivered in two fractions one week apart, resulting in a control rate of 85.5% [97]. Lin et al. reported a control rate of 91.3% for helical tomotherapy using a dose of 13.5 Gy, delivered in three fractions [2].

EBRT: High-Energy Photon Radiotherapy and Adverse Effects

Malaker et al. reported the occurrence of acute perichondritis in 6.3% of patients within one week post-radiotherapy, with only one patient requiring steroid treatment [96]. Lin et al. reported the occurrence of skin hyperpigmentation in all patients, which resolved within one year. Additionally, one patient with an earlobe keloid experienced mild hair loss around the irradiated area [2].

EBRT: Low-Energy Photon

Low-energy photon radiotherapy, encompassing kilovoltage to orthovoltage ranges, exhibits a dose deposition pattern similar to that of electron radiotherapy. A key advantage of low-energy photon radiotherapy is its ability to deliver the full prescribed dose directly to the skin surface without requiring additional bolus or wax. Furthermore, unintended exposure to surrounding tissue can be effectively minimized using simple, hand-made lead shielding prepared within a few minutes, as opposed to the Cerrobend blocks typically used in electron beam radiotherapy, which require several hours to fabricate.

EBRT: Low-Energy Photon, Dose, and Recurrence Rate

The reported recurrence rates of low-energy photon irradiation vary from 0% to 45.8% [89,98-105]. The dose regimens and associated recurrence rates are shown in Table 7. Yang et al. delivered 4-5 Gy × 2 fractions (on postoperative days 0 and 3) using the INTRABEAM system at a reference depth of 0 mm. A recurrence rate of 0% was reported within a median follow-up of 22.35 months (range: 15-32 months) [89]. Sakamoto et al. reported recurrence rates of 11% at a dose of >20 Gy delivered in five fractions, 18% at a dose of 20 Gy in five fractions, and 43% at a dose of <20 Gy in five fractions [98]. Speranza et al. administered 15 Gy in three fractions, reporting a recurrence rate of 45.8% based on scar descriptions collected via patient questionnaires; however, the nature of the reported scars was not documented, and they may represent residual surgical scars rather than true keloid relapse [100]. Mohamed et al. reported a two-year recurrence-free rate of 92.0 ± 4% following orthovoltage photon beam treatment in patients with ear keloids [103]. Recalcati et al. administered various dose regimens to patients with auricular keloid using contact X-ray (55-60 kV) and superficial X-ray (50 kV) therapy, achieving a recurrence rate of 13.2% [104].

**Table 7:** Reported recurrence rates according to radiotherapy regimens using low-energy photon team therapy. Annotation markers: ^b^ Inconsistency of estimated effective treatment efficacy between BED10 and BED2. BED, biologically effective dose; BED2, biologically effective dose calculated with α/β ratio 2 Gy; BED10, biologically effective dose calculated with α/β ratio 10 Gy

Fraction size	1 fraction	2 fractions	3 fractions	4 fractions	5 fractions
3 Gy	-	-	-	-	0% [101], 0% [102]
4 Gy	-	-	16% [99]	-	^b^ 18% [98]
8 Gy	6.25% [105]	-	-	-	-

EBRT: Low-Energy Photon and Adverse Effects

Compared to other radiotherapy modalities for keloid treatment, low-energy photon irradiation is associated with a higher incidence of skin reactions, including erythema and pigmentation changes. The reported acute adverse effects include erythema (36%-67%) [99,100,103], moderate skin redness (9%-42.3%) [100,103], severe redness (17.3%) [100], desquamation (12%-24%) [99,100], mild and short-term skin pruritus in up to 75% of patients [89,105], wound dehiscence (10.4%) [100], epidermolysis (3.2%) [104], and infection (6.3%) [100]. The documented late adverse effects included hyper- or hypopigmentation in 33.3%-100% [99,100,102,105,106], and telangiectasia in 27%-32.1% [99,100].

**Figure 5:**
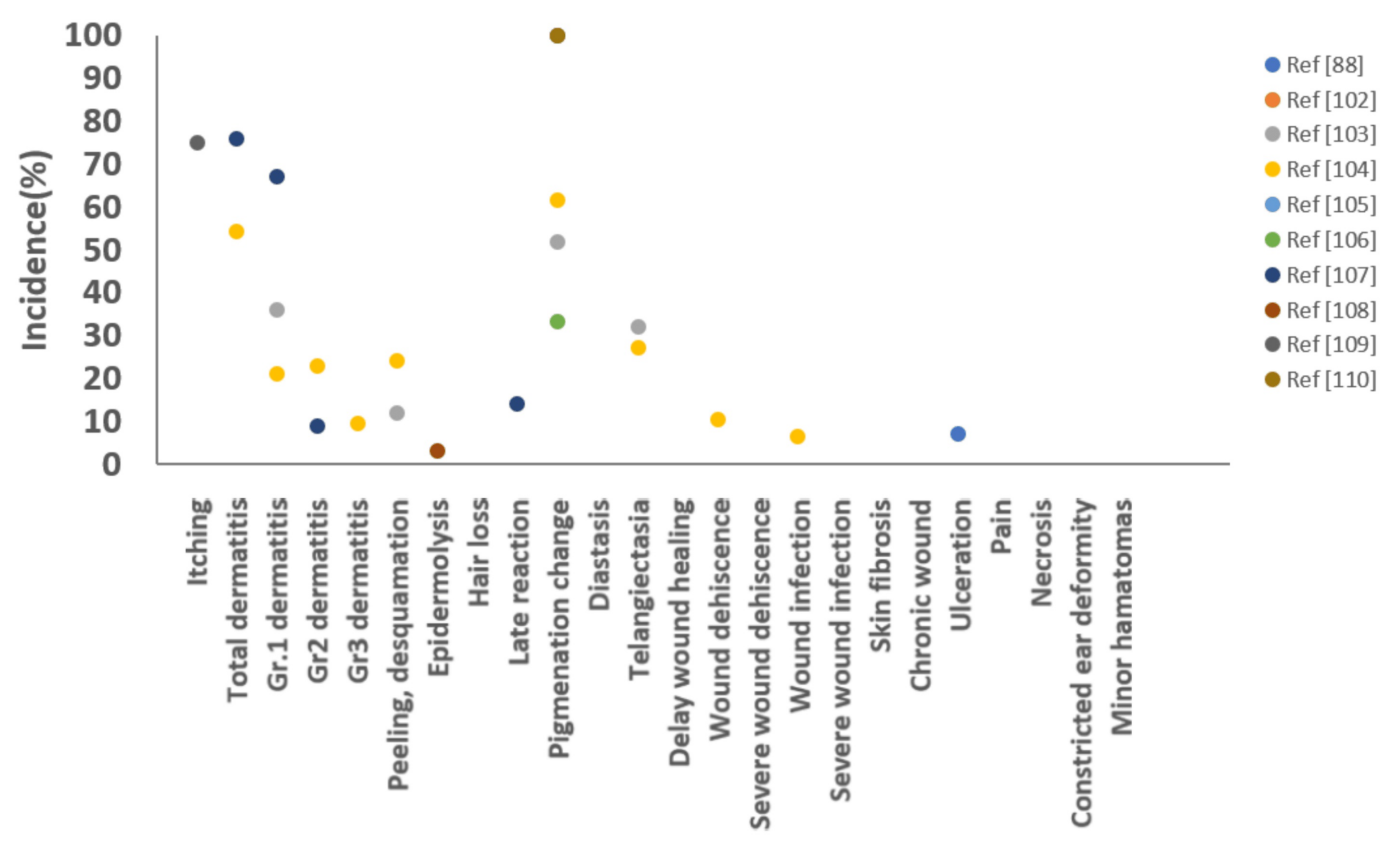
Incidence rates of adverse effects following low-energy photon beam radiotherapy reported in literature over the past 20 years. Each data point represents the incidence rate of adverse effects reported in an individual study.

The adverse effect rates from each low-energy photon radiotherapy study are summarized in Figure 5.

Discussion

Direct comparisons of treatment efficacy among different radiotherapy modalities are limited, primarily due to variations in institutional machine settings. The most commonly employed techniques include HDR interstitial brachytherapy, electron beam radiotherapy, and low-energy photon-beam irradiation. Table 8 provides a comparison of the advantages and disadvantages of various radiation modalities used for keloid treatment.

**Table 8:** Comparison of radiation dose distribution across different radiation modalities. CT, computed tomography

Radiation modality type/dose distribution	Disadvantage	Advantage
Interstitial brachytherapy	Require cooperation with surgeons to place the catheters; Require an extra brachytherapy room and machine; Not suitable for inoperable keloid; Higher rate of wound complications	Provides more uniform dose distribution along the wound contour; Treatment efficacy is well-supported by numerous clinical studies
Surface mode brachytherapy	Requires a dedicated brachytherapy room and a specialized machine; Limited number of studies available; Higher incidence of skin reactions	Irradiation field can be visually confirmed through direct contact with the treatment site
Electron radiotherapy	Requires several hours for electron cutout preparation; Dose distribution can be affected by body curvature	Does not require additional treatment room or specialized machine; Treatment efficacy is well supported by numerous studies; Suitable for institutions with limited resources
Low-energy photon radiotherapy	Limited number of studies; High incidence of skin reactions	Irradiation field can be visually confirmed through direct contact with the treatment site; Lead sheet block preparation takes only a few minutes; Portable machine
High-energy photon radiotherapy	Limited number of studies; Requires CT simulation; Requires a few hours for treatment planning; Potential for significant dose to deep critical organs	No need for additional treatment room or specialized equipment; Enables complex dose distribution through planning, suitable for complex keloids

Liao et al. compared the dose distributions of HDR interstitial brachytherapy and electron EBRT for the management of complex keloids. Their findings demonstrated superior dose coverage with HDR interstitial brachytherapy, suggesting its suitability for anatomically intricate lesions [107]. Yang et al. compared patients treated with 50 kV INTRABEAM (8-10 Gy in two fractions) and those treated with 6 MeV electron beam radiotherapy (12 Gy in three fractions delivered on non-consecutive postoperative days). A lower recurrence rate was observed in the INTRABEAM group than in the electron beam group [89]. Flickinger developed dose-response curves for various radiotherapy modalities. Their analysis indicated that linear accelerator (LINAC) electron beam and 60Co radiotherapy were associated with lower recurrence rates compared with 50-120 kV photon irradiation, potentially due to deeper tissue penetration and more effective dose distribution [38].

MV photon radiotherapy, referred to as high-energy photon, can also be applied to superficial skin lesions using techniques such as total skin irradiation with complex tangential beam arrangements [108]. Malaker et al. successfully treated earlobe keloids using a 60Co teletherapy unit. To minimize radiation exposure to adjacent organs, the ears were positioned vertically upward, while the neck and cheeks were aligned horizontally [96]. Telecobalt machines have also been utilized for treating keloids at other anatomical sites [97].

Based on this assumption, Eaton et al. aimed to improve the dose homogeneity for earlobe keloid treatment using a parallel opposed-pair approach to enhance disease control [109]. Mankowski et al. compared the treatment efficacies of different radiotherapy modalities. This study demonstrated that each modality has a unique BED-recurrence rate curve based on biological dose. Both electron therapy and brachytherapy were shown to achieve superior control rates compared with X-ray therapy. However, no significant difference was found in the recurrence rate between electron therapy and brachytherapy [110]. Conversely, Mohamed et al. compared the treatment efficacy between the orthovoltage photon beam and EBRT. Their results suggested a marginally higher two-year recurrence-free rate in the orthovoltage group (p = 0.09) [103].

Okuhata et al. conducted a phantom study demonstrating the feasibility of tomotherapy as an alternative modality for keloid treatment [111]. Compared with photon beams, electron beam radiotherapy tends to deliver inhomogeneous doses on irregular surfaces and at field junctions, which may contribute to higher recurrence rates [112-114]. Lin et al. reported that helical tomotherapy delivered surface doses of 103.7%-112.5% of the prescribed dose, whereas electron beam radiotherapy achieved 92.8%-97.6% [2]. With appropriate lesion selection and treatment planning, high-energy photon therapy offers a more homogeneous distribution and may serve as an effective alternative for keloid treatment.

**Figure 6:**
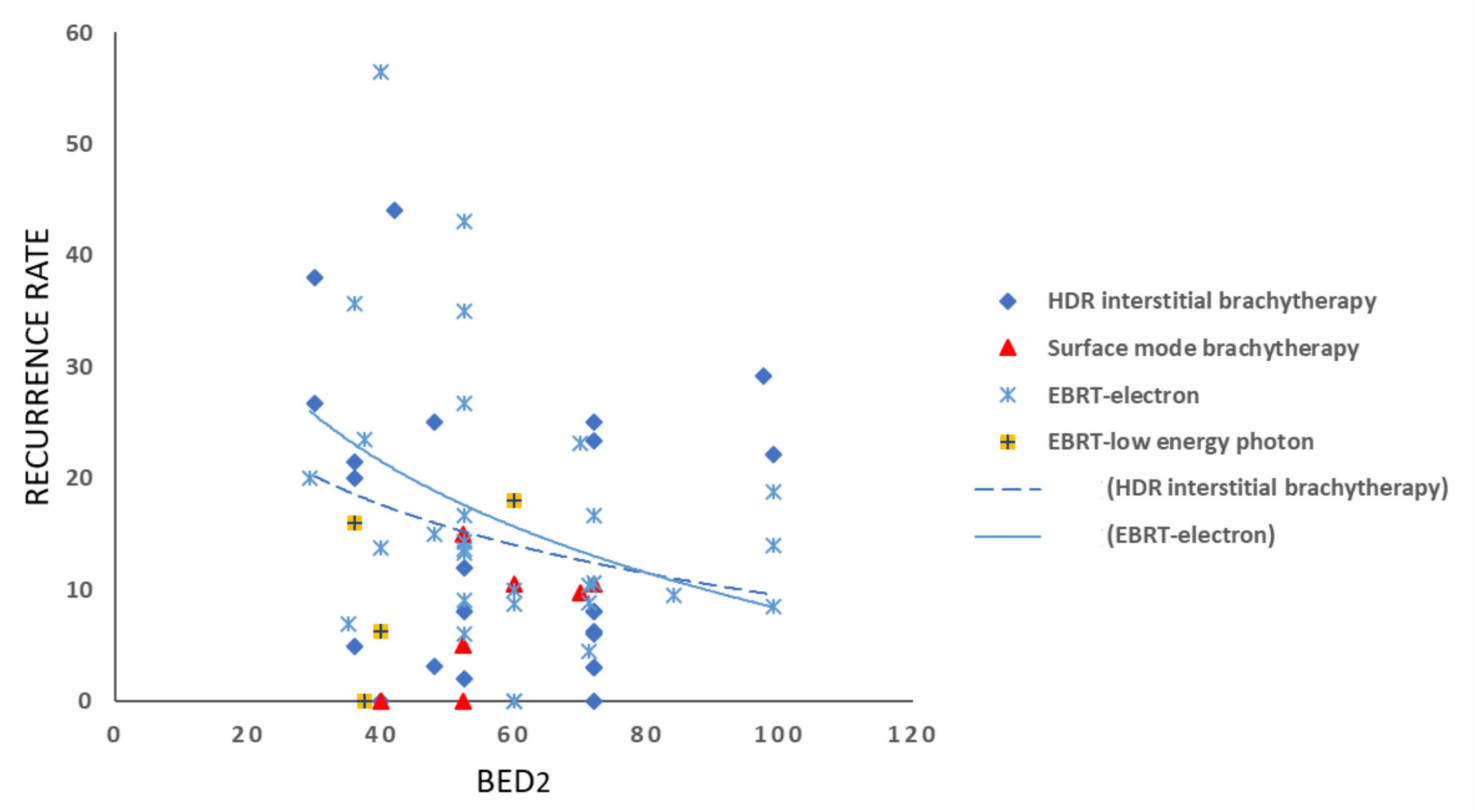
Summary plots of reported recurrence rates according to BED and modality type. Recurrence rates, based on radiotherapy modality and BED calculated with an α/β ratio of 2 Gy (late-responding tissue), are shown. Trend lines for HDR and electron radiotherapy illustrate treatment effectiveness. BED, biologically effective dose; HDR, high-dose-rate; EBRT, external beam radiotherapy

**Figure 7:**
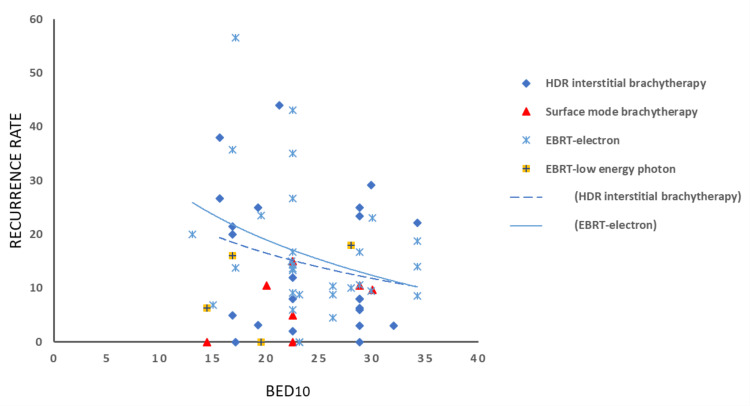
Summary plots of reported recurrence rates according to BED and modality type. Recurrence rate based on radiotherapy modality and BED, calculated with an α/β ratio of 10 Gy (early-responding tissue). Trend lines for HDR and electron radiotherapy. BED, biologically effective dose; HDR, high-dose-rate; EBRT, external beam radiotherapy

Conflicting conclusions have been drawn from studies comparing radiotherapy modalities for keloid treatment. The present review and recurrence summary plots (Figures 6-7) revealed a relatively low recurrence rate (0%-18%) associated with low-energy photon beam therapy; however, the available literature remains limited. In the lower BED range, the trend lines suggest that HDR radiotherapy offers superior control compared to electron radiotherapy. At target BED values (BED10 = 30 and BED2 = 60-70), both modalities appear to demonstrate comparable efficacy. Treatment outcomes are influenced by several factors beyond the radiotherapy regimen, including keloid location, timing between surgery and radiotherapy, use of consecutive versus interrupted fractionation, inter-fraction intervals, and technical considerations such as bolus and margin expansion. These variables must be taken into account when interpreting study findings.

Few studies have directly compared HDR and LDR brachytherapy for keloid treatment. According to Arnault et al., LDR brachytherapy did not show superior local control compared with HDR interstitial brachytherapy [67]. De Cicco et al. compared LDR brachytherapy, with a median dose of 16 Gy, with HDR brachytherapy, with a median dose of 12 Gy. Their findings showed improved symptomatic relief with HDR treatment (92.0% vs. 68.0%) [49]. In terms of late toxicities, the rates of hyperpigmentation and skin fibrosis were reported as 10.8% versus 22.0% and 32.6% versus 22.0% for LDR and HDR, respectively. Skin telangiectasia was observed in 15.2% of patients treated with LDR. The reported recurrence rates of HDR and LDR were 38% and 30.4%, respectively. However, the respective BED2.08 values of HDR and LDR were 29.3 and 22.3 Gy, respectively. Moreover, the BED10 values of HDR and LDR were 17.3 and 9.1 Gy, respectively. These values were below the recommended therapeutic thresholds, which may have contributed to the high recurrence rate [49]. Interestingly, van Leeuwen et al. reported that HDR interstitial brachytherapy achieved the lowest mean recurrence rate of 10.5 ± 15.0%, compared with 21.3 ± 2.1% for LDR interstitial brachytherapy, despite the higher dose in the LDR interstitial brachytherapy [115].

**Figure 8:**
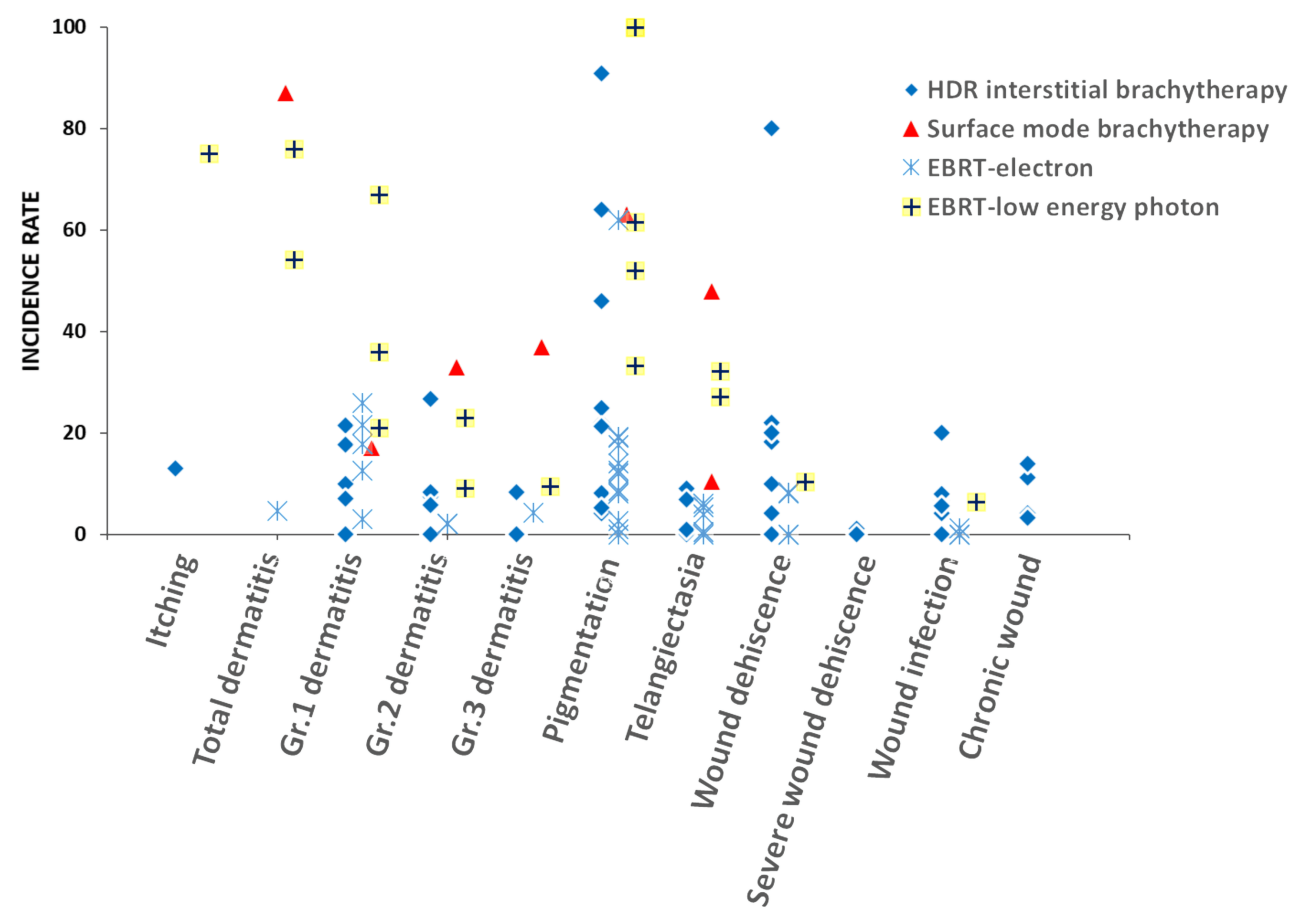
Summary plots of major adverse effect incidence rates according to different radiation modalities reported in the past 20 years. Each data point represents the incidence rate of adverse effects reported in an individual study. EBRT, external beam radiotherapy; HDR, high-dose-rate

Our review and adverse effect summary plots (Figure 8) reveal that surface mold brachytherapy and low-energy photon EBRT tend to lead to an increased risk of skin-related side effects, including itching, dermatitis, and pigmentation changes. By contrast, HDR interstitial brachytherapy is associated with a higher rate of wound dehiscence compared with electron EBRT or low-energy photon EBRT. Considering that HDR interstitial radiotherapy directly irradiates the wound bed, a greater impact on wound healing is expected. Among the modalities, electron EBRT generally demonstrates the lowest incidence of adverse effects. Table 9 compares recurrence rates, adverse effects, and the limited number of references available for each treatment modality.

**Table 9:** Comparison of recurrence rates, adverse effects, and available literature across different radiation modalities for keloid treatment.

Modality	Recurrence rate	Adverse effects	Reference count
Interstitial brachytherapy	Treatment efficacy well-supported by clinical studies	High incidence of pigmentation and wound complications	17 available studies
Surface mode brachytherapy	Relative lower documented recurrence rate	High incidence of dermatitis, pigmentation, and telangiectasia	Limited references: 6 available studies
Electron radiotherapy	Treatment efficacy well supported by clinical studies	General lower incidence of adverse effects compared to other treatment modalities	29 available studies
Low-energy photon radiotherapy	Relative lower documented recurrence rate	High incidence of dermatitis and pigmentation	Limited references: 10 available studies

Most recurrences occur within the first year post-treatment [64,74,79], with only 15% occurring after three years, as reported by Viani et al. [65]. Numerous studies have demonstrated that recurrence rates vary across radiation regimens and are strongly dose-dependent [73,98]. BED10 = 30 or BED2 = 60-70 has been proposed as a predictive threshold for treatment efficacy. A higher BED has been consistently associated with a lower recurrence rate [36,39]. Keloid control may be improved by increasing both the total radiation dose and fraction size [36-38]. Duan et al. demonstrated that hypofractionated radiotherapy provides significantly better control compared with conventional fractionation, supporting the influence of fraction size on treatment outcomes [116].

Radiotherapy doses should be tailored to the keloid’s location and size. Sreelesh et al. administered adjuvant electron beam radiotherapy at 8 Gy in a single fraction, reporting an overall recurrence rate of 56.5%. However, a sub-analysis revealed a significantly lower recurrence rate of 8.3% for earlobe keloids. Based on these findings, an 8 Gy single fraction appears appropriate for earlobe keloids, whereas higher doses may be required for keloids located in higher-tension areas [74]. Similarly, Mohamed et al. found that single-fraction doses of 8 and 13 Gy yielded significantly different local control rates for keloids >2 cm, but not for those <2 cm [103]. This result suggests that a larger keloid may require a higher irradiation dose for optimal control.

Sruthi et al. reported recurrence rates of 16.2% in patients treated with 5-12 Gy in one to three fractions, and 13.8% in those receiving 8 Gy in a single fraction [82]. Hsueh et al. administered 5 Gy × 4 fractions to the anterior chest wall, scapular region, suprapubic region, and mandible, and 5 Gy × 3 fractions to other regions. Radiotherapy was initiated on the day of surgery in 36.7% of patients, and at two weeks postoperatively in 63.3% of patients, with an overall recurrence rate of 32% [29]. In a recent report, Ogawa et al. recommended 8 Gy × 1 fraction for earlobe lesions; 6 Gy × 3 fractions for high-recurrence regions, including the anterior chest wall, scapular region, and suprapubic region; and 7.5 Gy × 2 fractions for lesions at other anatomical sites, based on clinical experience [73].

In addition to radiotherapy regimens, several factors affecting keloid control rates have been investigated. These include sex (both female sex [98] and male sex [44,49,69,74,78]), younger age [49,69,78], high-tension anatomical sites [36,49,56,65,69,70,74,78,93], larger lesion size [65,69,70,74,103], multiple lesions [91,94], and longer intervals between surgery and radiotherapy [70,76,93,115]. Additional risk factors associated with increased recurrence include spontaneous keloid etiology [56,93], burn-induced keloids [65], symptomatic keloids [49], inadequate radiation field margins [105], graft reconstruction [69], and prior treatments [65,70]. Furthermore, a delay of more than 4.2 years between keloid onset and subsequent surgery followed by radiotherapy has also been proposed as a significant risk factor for recurrence [79].

Several studies have reported that wound infections and chronic wounds are associated with increased recurrence rates [41,51]. Liu et al. identified wound infection as a significant risk factor for local recurrence [70]. In addition, atypical keloid locations with inhomogeneous radiotherapy dose distributions may contribute to treatment failure [57]. The complexity of electron beam radiotherapy dose distribution - affected by the skin-sparing effect, field shrinkage, uneven surfaces, and small treatment areas - poses further challenges. Therefore, careful adjustment of radiotherapy settings based on modality and anatomical considerations is essential to optimize treatment outcomes. Table 10 lists the potential factors that may influence treatment outcomes during radiotherapy, including treatment modalities, radiation dose and fractionation schedules, as well as technical parameters such as beam energy and targeting accuracy. Understanding these factors is crucial for optimizing therapeutic efficacy and minimizing adverse effects.

**Table 10:** Factors influencing outcomes during radiotherapy.

Factors influencing outcomes during radiotherapy	
Dose protocols	Total dose, fractionation schedule
Treatment apparatus	Interstitial brachytherapy, surface mode brachytherapy, external beam radiotherapy
Radiation type	Radioactive source, photon, electron
Technical parameters	High energy, low energy
Clinical settings	Field design, field size, bolus

Surgical technique has also been identified as a key factor in improving keloid control rates. Ogawa et al. suggested that tension-reducing techniques, including subcutaneous and deep fascial tensile reduction sutures and Z-plasty, significantly reduce keloid recurrence [73,81,94,117,118]. For large keloids, particularly in challenging locations such as the auricular cartilage, where complete resection and closure are difficult, core excision followed by simple suturing and adjuvant radiotherapy markedly reduced the recurrence rate from 21.4% to 9.4%, despite using the same radiotherapy regimen [73,119]. Wang et al. proposed a subcutaneous super-tension reduction suture combined with electron beam irradiation of 5 Gy for three to four consecutive days, initiated within 24 hours postoperatively. A low relapse rate of 2.2% (1/45) for chest keloids was reported at the two-year follow-up [88].

Park introduced a triple combination therapy consisting of 2-mm punch biopsy-assisted partial excision, intralesional triamcinolone injection, and a single fraction of 10-11 Gy external beam radiation therapy. The 2-mm punch biopsy-assisted partial excision was designed to reduce the mechanical force of internal and surrounding tissues through a minimally invasive approach. In the study, no aggravation or recurrence was observed during the 12-month follow-up among 30 patients who were unsuitable for complete excision or primary closure after surgery [120].

Patients in the Ogawa et al. group underwent Z-plasty followed by 6 Gy in three fractions of electron beam radiation, combined with salvage treatment using steroid plaster therapy. The recurrence rates were reported as 10.6% for keloids associated with folliculitis/acne and 5.3% for keloids resulting from small injuries caused by Bacillus Calmette-Guérin vaccination [81,94]. Shen et al.'s group reported a relatively low recurrence rate of 8.6% at a median follow-up of 22 months following treatment with Z-plasty and immediate radiotherapy administered within four hours, using a relatively lower dose of 12-16 Gy delivered in four daily fractions [87].

Details of the risk factors for recurrence are presented in Table 11.

**Table 11:** Risk factors for increase recurrence rate. OP: operation; RT: radiotherapy; BED: biologically effective dose

	Gender	Younger age	Etiology	Keloid size	Characteristic of keloid	Location	OP factors	OP-RT factors	RT factors	Wound factors	Other factors
Sakamoto et al. (2009) [98]	Female	-	-	-	-	-	-	-	Total dose of <20 Gy	-	-
Shen et al. (2015) [69]	Male	£ 29	-	>5 cm	-	High stretch tension: chest wall, back, shoulder, and lower limbs	With skin graft	Longer OP-RT interval	-	-	-
Bijlard et al. (2018) [44]	Male	-	-	-	-	-	-	-	-	-	-
De Cicco et al. (2014) [49]	Male	<44	-	-	Symptomatic keloid	Arms, neck, chest wall	-	-	-	-	-
Katano et al. (2024) [78]	Male	<50	-	-	-	Chest wall, shoulder, and suprapubic region	-	-	-	-	-
Kal and Veen (2005) [36]	-	-	-	-	-	High stretch tension such as thorax	-	-	Less BED	-	-
Franzetti et al. (2024) [56]	-	-	Spontaneous keloid	-	-	Sternum and limbs	-	-	-	-	-
Liu (2023) [70]	-	-	-	Maximum diameter	-	-	-	Longer OP-RT interval	-	Infection	Previous treatment
Sreelesh et al. (2023) [74]	Male	-	-	>5 cm	-	Location other than earlobe	-	-	-	-	-
Wen et al. (2021) [93]	-	-	Spontaneous keloid	-	-	Trunk and limbs	-	Longer OP-RT interval	-	-	-
Viani et al. (2009) [65]	-	-	Keloid from burn	>5 cm	-	Thorax	-	-	-	-	Previous treatment
Mohamed et al. (2022) [103]	-	-	-	>2 cm	-	-	-	-	8 Gy for keloids exceeding 2 cm	-	-
Dohi et al. (2020) [91]	-	-	-	-	Multiple keloids	-	-	-	-	-	-
Dohi et al. (2019) [94]	-	-	-	-	Multiple keloids	-	-	-	-	-	-
Lee and Park (2015) [76]	-	-	-	-	-	Shoulder, chest wall, abdomen	-	-	-	-	-
Carvajal et al. (2016) [79]	-	-	-	-	Symptomatic keloid	-	-	-	-	-	Interval from diagnosis to OP-RT of >4.2 years

The risk factors for adverse effects from superficial X-ray therapy have been identified as keloid location, incision length exceeding 5 cm [106], previous treatment, and radiation dose [98]. In Li et al.’s study, hyperpigmentation rates reached 100% in trunk keloids and 92.89% in limb keloids. Meanwhile, head and face keloids exhibited significantly lower rates of severe pigmentation [106]. In Sakamoto et al.’s study, the adverse effect rates were 44% with total doses exceeding 20 Gy delivered in five fractions, 18% at 20 Gy in five fractions, and 7% at doses below 20 Gy. Their analysis also identified older age, minor etiology, and prior treatment as factors associated with increased adverse effects [98].

Radiotherapy alone can be a treatment option for patients with large keloids or comorbidities that preclude surgery. For unresectable lesions, surface mode brachytherapy and EBRT are commonly used. Yang et al. irradiated two inoperable patients with 10 Gy delivered in two fractions at a reference depth of 0.5 cm below the skin. One patient developed a superficial ulcer one month after treatment [89]. Sari et al. administered 37.5 Gy in five fractions, primarily via electron beam irradiation, to six patients with 13 keloids. Over 12-21 months of follow-up, complete remission was observed in all patients within three to six months after radiotherapy. The adverse effects included erythema and dry desquamation in 100% of patients; Grade 2 dermatitis in 100%, all of which resolved completely within six months; ulceration in 66.7%; epilation in 33.3%; pigmentation changes in 50%; and fibrosis in 33.3% [121]. Malaker et al. applied the same regimen of 37.5 Gy in five fractions to 86 unresectable keloids in 64 patients. They reported that 97% of lesions showed significant regression 18 months after radiation, with acceptable adverse reactions [122].

To increase the efficacy of radiotherapy for nonoperable keloids, combination therapies involving multiple treatment modalities have been evaluated. Liu et al. demonstrated a cocktail regimen comprising pulsed dye laser therapy, injections of triamcinolone acetonide (TAC) + fluorouracil (FU), sodium chloride + FU injection, and electron radiotherapy - 18 Gy in two fractions - followed by FU injections. This approach was designed to enhance the overall effectiveness of TAC and radiotherapy, with FU used as a radiosensitizer. Among the 80 patients treated, 45% completed the full course, and treatment efficacy was reported as 100%, with a cure rate of 92.8% [123]. Radiotherapy thus represents an effective option for managing unresectable keloids by alleviating symptoms and promoting lesion regression. For patients who are unsuitable candidates for radiotherapy, minimally invasive alternatives have also been investigated. Cryotherapy, either applied alone or combined with surgical approaches, has shown potential benefits. Punch excision followed by immediate cryotherapy has been reported to result in a significant reduction in VSS scores, potentially improving treatment efficacy by reducing mechanical tension [124].

Limited evidence is available regarding repeated irradiation for keloid treatment. Although a few patients have undergone second courses of radiotherapy in various studies, Grade 3 complications have been infrequently reported [53,57]. Garg et al. treated 17 patients with recurrent keloids using adjuvant 192Ir HDR brachytherapy. All 12 patients had previously received external beam electron radiotherapy (6-9 MeV) at doses ranging from 12 to 15 Gy in three fractions. A subsequent dose of 15 Gy in three fractions was administered postoperatively as adjuvant radiotherapy. A local control rate of 88% was achieved at a median follow-up of 26 months. The reported adverse effects included Grade 3 acute skin toxicity in one patient (8.3%) and ulcerations over the sternum in two patients (16.6%), one of whom required conservative management [50]. Although re-irradiation may increase the risk of Grade 3 adverse effects, it remains a safe and acceptable option for recurrent keloids when combined with surgical excision and supported by meticulous wound care.

## Conclusions

Keloids are benign skin lesions, where achieving an optimal balance between effective control and minimizing adverse effects is essential. Combined treatment with surgery followed by radiotherapy provides superior local control of keloids compared to monotherapy. Several factors influence radiotherapy distribution and treatment outcomes, including dose protocols, treatment equipment, radiation source type, radiation energy levels, and clinical settings. Currently, HDR interstitial brachytherapy and electron EBRT are the primary modalities used for keloid treatment, both demonstrating satisfactory control rates. Although low-energy photon EBRT and surface mode brachytherapy appear to be associated with low recurrence rates, available data remain limited. Treatment strategies should be tailored according to institutional resources and modality-specific considerations. Effective collaboration and communication between surgeons and radiation oncologists are vital to optimizing treatment outcomes. Further exploration of each modality and follow-up results is warranted.
